# Detection of Nonylphenol with a Gold-Nanoparticle-Based Small-Molecule Sensing System Using an ssDNA Aptamer

**DOI:** 10.3390/ijms21010208

**Published:** 2019-12-27

**Authors:** A-Ru Kim, Sang-Heon Kim, Dabin Kim, Seo Won Cho, Ahjeong Son, Moon-Young Yoon

**Affiliations:** 1 Department of Chemistry and Research Institute of Natural Sciences, Hanyang University, Seoul 04763, Korea; kimr2122@hanyang.ac.kr (A.-R.K.); konasi2@naver.com (S.-H.K.); 2 Department of Environmental Science and Engineering, Ewha Womans University, Seoul 03760, Korea; dabin_kim@naver.com (D.K.); 94petite@naver.com (S.W.C.); ason@ewha.ac.kr (A.S.)

**Keywords:** endocrine disruptors, nonylphenol, SELEX, aptamer, reduced graphene oxide, detergent

## Abstract

Endocrine-disrupting chemicals (EDCs) threaten many kinds of life throughout the world. These compounds function the same as sexual hormones, inducing precocious puberty, gynecomastia, etc., in the human body. To prevent excess exposure to nonylphenol (NP), a simple and rapid detection system is needed. In this study, we develop a nonylphenol-specific aptamer from a random single-stranded DNA library and test a rapid sensor system based on the aptamer and gold nanoparticles (AuNPs). The aptamer was screened by a methodology involving reduced graphene oxide (rGO). As a result of screening and sequencing, a DNA aptamer was developed that recognizes the target with high binding affinity (K_d_ = 194.2 ± 65.9 nM) and specificity. The sensor system developed using the aptamer and gold nanoparticles is sensitive (LOD = 2.239 nM). Circular dichroism (CD) spectrometry results show that the free aptamer binds to the target molecule. The aptamer was characterized using gold nanoparticles to measure UV absorbance. Our results suggest that the sensor system developed using this aptamer is useful for field diagnosis of small molecules.

## 1. Introduction

Many petrochemicals are used in production of various products that add quality and convenience to our lives. However, some of these chemicals affect the human body and may disrupt the reproduction process or cause precocious puberty and other disorders of the reproductive system. Generally, these materials are called endocrine-disrupting chemicals (EDCs) and are exogenous substances or mixtures that alter the functions of the endocrine system and consequently cause adverse effects in an intact organism, its progeny, or populations [1,2]. Sustained exposure to EDCs can cause many diseases, such as cancers, obesity, diabetes, and sterility. Because some kinds of EDCs are similar to sexual hormones, they can bind to sexual hormone receptors.

Nonylphenol is one of the chemicals that can affect the human endocrine system. It has been shown to mimic the natural hormone 17β-estradiol and competes with the endogenous hormone in binding with the estrogen receptors ERα and ERβ [3]. Nonylphenol is used as an additive in antioxidants, lubricating oil additives, laundry and dish detergents, emulsifiers, and solubilizers [3] and is used in polystyrenes to stabilize plastic food packaging.

There are several methods for detecting nonylphenol. Systems based on immunology are easy and fast. Most use antibodies that bind to the target with high binding affinity and specificity. However, this type of system also has weaknesses, the most serious being that antibodies are proteins. Proteins have very complicated biomolecular structures that are easily affected by changes in the environment, so the storage conditions of antibody-based systems must be managed very carefully.

To solve this problem, many ideas for replacement of antibodies have been proposed, including use of aptamers. An aptamer is short single-stranded nucleic acid (DNA or RNA) molecule that also binds to a target with specificity and binding affinity. As aptamers are much smaller biological molecules than antibodies, they have various positive attributes, including simple synthesis and chemical modification, as well as low immunogenicity [4,5,6,7]. Aptamers can be screened against targets through systematic evolution of ligands using the exponential enrichment (SELEX) method [7,8]. The SELEX method is widely used to identify aptamers that bind to various targets, including small molecules, proteins, cells, tissues, and organisms [9,10].

Traditional SELEX methods require a target binding step, which can be a difficulty. For instance, if the target chemical is bound to a plate or bead, it is not an intact molecule. Also, it is difficult to immobilize some kinds of molecules. To overcome these problems, our SELEX process used reduced graphene oxide (rGO) to screen small molecules based on prior research [11]. Six carbons composed a ring structure within the graphene that was able to interact with the ring structure of the oligonucleotide through π-π interactions [11,12]. A single-stranded nucleic acid was able to bind to the graphene sheets. To separate this complex, some methods require addition of a small molecule or protein, a high concentration of salt, etc. to induce a conformational change or complementary sequences. These methods make use of the resulting interaction between the single-stranded oligonucleotide and rGO of the exposed phosphate backbone [11,12,13,14].

Gold nanoparticles are commonly used in colorimetric diagnostic systems because aggregation of these particles is controlled by salt. The influence of size and shape of AuNPs used in colorimetric assays has been investigated [15]. However, aptamers prevent the influence of salt on aggregation of gold nanoparticles [16].

Herein, we describe the development of a specific aptamer sequence and suggest a simple sensor system based on this aptamer and gold nanoparticles. Briefly, we screened the aptamer using rGO against an immobilized target that was then characterized with gold nanoparticles to show the usefulness of our system as a simple and convenient test kit.

## 2. Results

### 2.1. Screening Aptamer Candidates against Nonylphenol via rGO-SELEX

Graphene oxide and reduced graphene oxide can settle down more easily than graphene. Non-bound ssDNAs were eliminated by centrifugation. The ssDNAs adsorbed on the rGO surface were eluted through treatment with the target compound. This operation was repeated for five rounds. However, in the fourth round, some of the ssDNA bound on the rGO was eluted through treatment with counter target compounds (bisphenol A (BPA), phenyl phenol (PP), phenol, and diethylhexyl phthalate (DEHP)) and eliminated by centrifugation. Each subsequent round progressed with harsher buffer conditions and shorter elution times. These processes allowed us to maintain the ssDNAs with higher specificity to the original target compound.

**Figure 1:**
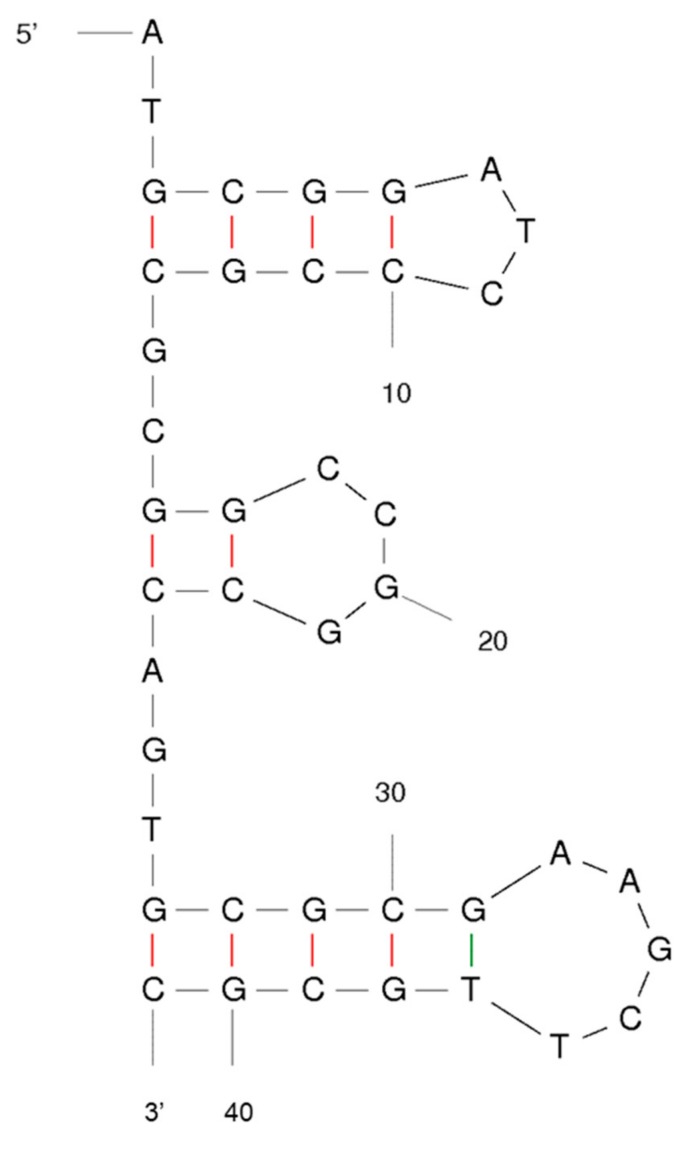
The secondary structures of candidate aptamer NP7.

Lastly, the eluted ssDNAs from the final round were amplified to confirm the sequence. The amplified oligonucleotides were inserted into the pET-28a vector and inoculated. From the *Escherichia coli (E. coli)* cultured at 37 °C overnight in LB medium, one selected sequence that confirmed repeatably was found and labeled. The selected aptamer structure was predicted by the M-fold program (Figure 1).

### 2.2. Binding Affinity of the Selected Aptamer to Nonylphenol

**Figure 2:**
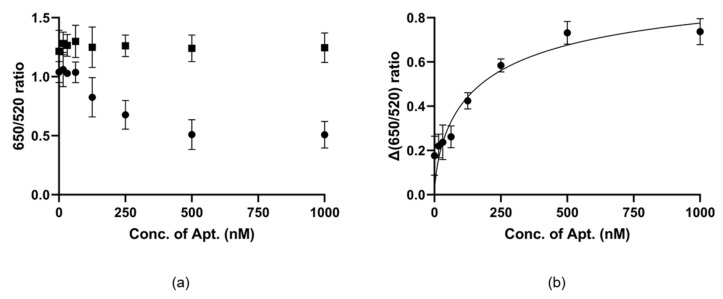
The binding affinity of NP7 in the AuNPs colorimetric assay. (**a**) UV absorbance ratio of control line which is without the target material (solid square, top) and test line which is with the target material (solid sphere, bottom). (**b**) Differences in UV absorbance ratio of control and test lines.

We made use of this characteristic to confirm the binding affinity. This experiment is done by comparing the control line without the target material (Figure 2a, solid square) with the test line with the target material (Figure 2a, solid sphere). When salt was added to these lines, the test line was completely aggregated and maintained nearly the same color, while the color of the control line changed with aptamer concentration after the reaction. For precise confirmation and quantification, we measured the ratio of absorbance (Figure 2). Figure 2a shows the ratio of the two lines, and Figure 2b shows the distance between the two lines and the binding affinity of NP7. The gap in ratios increased in proportion to the concentration of NP7, which reached saturation near 500 nM. This experimental process was repeated three times, and K_d_ (dissociation constant) was determined to be 194.2 ± 65.9 nM.

### 2.3. Specificity Test against Similar Chemicals

**Figure 3:**
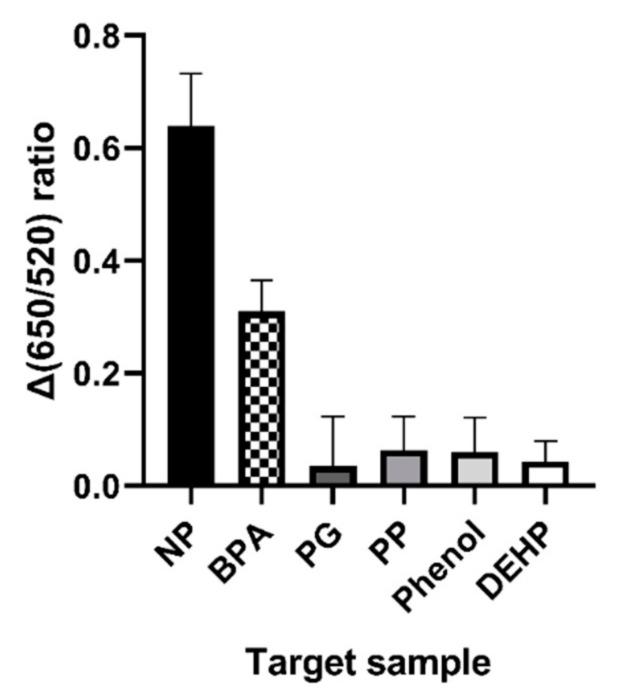
Specificity of NP7 against other endocrine-disrupting chemicals (EDCs) and similarly structured chemicals in the AuNP colorimetric assay. We used 6 endocrine-disrupting chemicals in here; nonylphenol (NP), bisphenol A (BPA), benzylpenicillin (penicillin G, PG), phenylphenol (PP), phenol, and Di-2-ethylhexyl phthalate (DEHP).

Other properties of NP7 were analyzed using the aforementioned characteristics of gold nanoparticles. As shown in Figure 3, NP7 had a higher specificity for the target substance than other compounds. The color of the gold nanoparticles changed when nonylphenol was added and only slightly when Bisphenol A (BPA) was added. The others produced results similar to those of the control. However, phenol and phenylphenol (PP) didn’t affect the aptamer.

### 2.4. Nonylphenol Detecting Test from Real Sample

**Figure 4:**
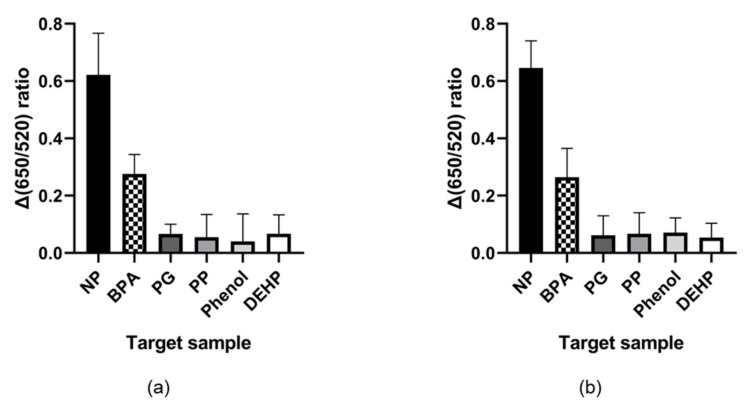
Specificity of NP7 against other EDCs and similarly structured chemicals in the AuNP colorimetric assay in (**a**) tap water and (**b**) 0.1% domestic detergent solution.

To confirm the detecting capability in real sample, we tested the specificity in tap water sample condition and 0.1% domestic detergent solution based on tap water. As shown in Figure 4, the results of this experiment also showed not much different from the existing conditions.

### 2.5. Conformational Change of Aptamer against Nonylphenol Addition

**Figure 5:**
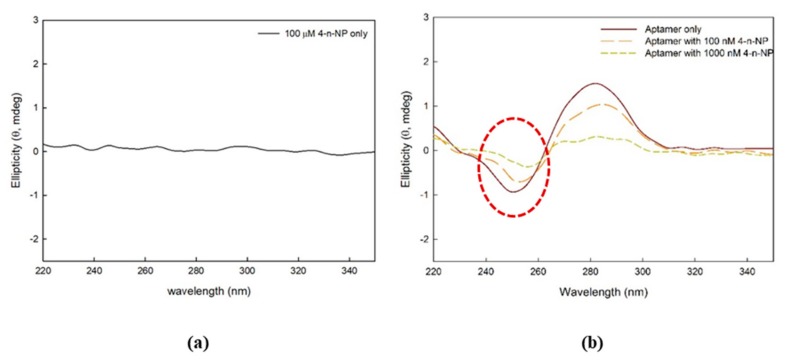
Ellipticity of (**a**) 4-n-NP only and (**b**) aptamer with varying concentrations of 4-n-NP. The red dotted circle in Figure 5b indicate interaction of 4-n-NP with the aptamer changed the CD spectrum at ~250 nm.

The conformational change of the aptamer was shown in the circular dichroism (CD) spectra when various concentrations of 4-n-NP were added in solution. When measuring the ellipticity of 4-n-NP without the aptamer (negative control), the value was close to zero, as shown in Figure 5a. This indicates that 4-n-NP alone does not change the CD spectrum. As shown by the red dotted circle in Figure 5b, interaction of 4-n-NP with the aptamer changed the CD spectrum at ~250 nm.

### 2.6. LOD Determination of NP7 Aptamer in the AuNP-Based Sensor System

The limit of detection (LOD) value of NP7 was determined by a process similar to the binding affinity test. A qualitative measure of the AuNP-based sensing system requires only one line to confirm the colorimetric change. However, we measured the ratio difference as a quantitative measure.

**Figure 6:**
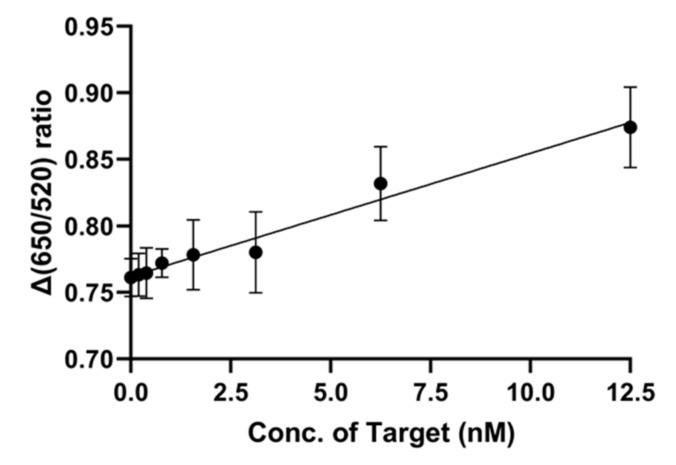
The detection limit of the AuNP-NP7 aptanano sensor system. LOD using the official formula from the International Union of Pure and Applied Chemistry (IUPAC) (LOD = 3 × SD/slope).

The method for determining LOD value is similar to those of the binding affinity and specificity tests mentioned above. We observed the color change of gold nanoparticles and measured the absorbance, which is dependent on the target molecule concentration. As the target molecule concentration increased, the color of the gold nanoparticles changed (Figure 6). According to the UV absorbance change in the range from 0 to 12.5 nM of nonylphenol, the LOD was determined to be 2.239 nM. The *R^2^* value was calculated to be 0.9752, which is indicative of an accurate result.

## 3. Discussion

Candidates able to bind to the nonylphenol were screened using rGO. It is known that ssDNA or RNA can bind to rGO via π–π stacking interactions between the polycyclic aromatic rings of the graphene surface and the nucleotides [11,12]. In the screening process, the nonylphenol molecule could also bind to the rGO because it has an aromatic ring structure. However, this phenomenon should be negligible. Because we used enough excess ssDNA to cover all rGO and the corresponding amount of target compound, it does not matter that the target binding aptamers eluted. According to the literature, the conformation of the aptamer changes after binding with the target and weakens the π–π stacking interactions with rGO [17,18,19,20].

Although we started with a 60 nt library with 30 random sequences, the selected sequence was only a 41 nt. This sequence is generally thought to be a PCR error when amplified for the next round [21] and is excluded from the candidates. However, this sequence was confirmed extraordinarily, leading us to select it.

Gold nanoparticles in distilled water are aggregated when salt is added [15]. However, an aptamer can interrupt such aggregation [16]. When an aptamer incubates with gold nanoparticles, it covers the particle surfaces. Then, when salt is added to a mixture of gold nanoparticles and aptamer, the nanoparticles are unaffected [16].

K_d_ was determined to be 194.2 ± 65.9 nM. This is better than values seen for several small-molecule-binding aptamers displaying affinities in the low- to mid-micromolar range [22,23]. Thus, the value of K_d_ in the nanomolar range suggests that NP7 has a high binding affinity for the target.

Specificity testing was performed with similar chemicals, some being structurally similar and others being similar in purpose and categorized as EDCs. Penicillin G, also known as benzylpenicillin, and DEHP have aromatic ring groups. Phenol, phenylphenol, and bisphenol A have phenyl ring groups. These chemicals are similar structurally, and bisphenol A and DEHP are used to change the properties of polymers. Considering only nonylphenol and bisphenol A from Figure 3, the result suggested that the phenyl ring group is the binding site of the aptamer. However, considering phenylphenol and phenol from the same figure, the phenyl ring group is not the only binding site. Taken together, it is thought that the phenyl ring group and some length of hydrocarbon branch act as binding sites. Finally, we also confirmed this result tap water and 0.1% domestic detergent solution based on tap water. In these conditions, the system can also detect specifically and almost same with in distilled water condition. From these results, we suggest that this system is not significantly affected by additional ingredient.

The spectral peaks in the negative band increased positively as the concentration of 4-n-NP increased. Even though the detailed mechanism needs to be elucidated, intercalation of 4-n-NP within the particular base pairs of the aptamer may have occurred when 4-n-NP was combined with the aptamer [24,25].

## 4. Materials and Methods

### 4.1. Materials

Nonylphenol was bought from Kanto Chemical (Tokyo, Japan). Primers and the template to produce the single-stranded DNA (ssDNA) library and amplify the elution product were synthesized by Bioneer (Daejeon, Korea). Pfu polymerase was purchased from Biofact (Daejeon, Korea), and restriction endonucleases and DNA ligases were obtained from Takara Bio (Shiga, Japan). Reduced graphene oxide (rGO) was purchased from the Graphene Supermarket (Calverton, NY, USA), and bisphenol A (BPA), phenyl phenol (PP), phenol, and diethylhexyl phthalate (DEHP) were obtained from Sigma-Aldrich (St. Louis, MO, USA). Penicillin G (PG) was obtained from Duchefa Biochemie (Haarlem, Netherlands). All other chemicals were obtained from commercial sources and were the highest quality available.

### 4.2. Construction of the ssDNA Library

The methodology used here is the same as that described in previous research [26]. A random ssDNA library was first used to find a specific binding aptamer for nonylphenol. A template for an ssDNA library containing 30 randomly-generated nucleotide sequences (5′-ATGCGGATCCCGCGC-(N)30-GCGCGAAGCTTGCGC-3′) in a total of 60 bases was used for this task. Forward primer, 5′-ATGCGGATCCCGCGC-3′ (containing a BamHI site), and reverse primer, 5′-GCGCAAGCTTCGCGC-3′ (including a HindIII site), were used to amplify the template DNA library using asymmetric PCR. A primer ratio of 10:1 was achieved using the same volume of each primer but with a forward primer concentration 10 times higher than that of reverse primer. After PCR, a few microliters of the product were electrophoresed on 2.5% agarose gel to confirm the product; the remainder was electrophoresed on a 12% native gel to separate double-stranded DNA (dsDNA) and ssDNA. The ssDNA library was isolated using the crush and soak method. After electrophoresis, the DNA was stained with ethidium bromide (EtBr). Then, the ssDNA band was cut out, the cut gel was pulverized, and the ssDNA was extracted using a crush and soak buffer (500 mM NH4OAc, 0.1% SDS, 0.1 mM EDTA).

### 4.3. Reduced Graphene-Oxide (rGO) SELEX against Nonylphenol

The methodology used here is the same as that described in previous research [26]. The aptamer specifically bound to nonylphenol was screened by the SELEX methodology using the ssDNA library. We performed a total of five rounds of screening, with the fourth round being a counter selection step in which a mixture of other chemicals with structures or uses similar to those of nonylphenol was tested.

The total volume used for the reaction was 200 µL and comprised 100 µL of water, 40 µL of 5× binding buffer (Tris-HCl 125 mM, NaCl 500 mM, KCl 125 mM, MgCl_2_ 12.5 mM, DMSO 25% (*v*/*v*)), 40 μL of rGO (5 mg/mL, diluted in water), and 250 picomoles of the ssDNA library. This mixture was reacted for 30 min to immobilize the ssDNA library onto the rGO. Then, the mixture was centrifuged at 14,000 rpm for 20 min, and the supernatant was discarded to eliminate any excess ssDNA that remained unbound to the rGO. The ssDNA-library-bound rGO pellet was washed one time with 200 µL of 1× binding buffer and centrifuged again under the same conditions to eliminate any unbound ssDNA. Forty nanomoles of nonylphenol diluted in 200 μL of 1× binding buffer was added to the pellet after the washing step. Nonylphenol and the rGO mixture were reacted for 1 h to elute the target binding aptamer from the rGO. The eluted candidates were amplified using the same methodology as was used for ssDNA library construction. A course of this series was counted as one round. The binding time and buffer concentration of each round were regulated to select a sequence that was specific to the target.

For the fourth round, the negative selection round, we added a step. After washing, we added the counter target mixture instead of nonylphenol. The counter target mixture volume was the same as that of the nonylphenol in the other rounds. The counter target mixture contained bisphenol A (BPA), diethylhexyl phthalate (DEHP), and phenyl phenol (PP) in the same ratio and total amount as the nonylphenol used in the positive selection rounds. After 1 h of reaction time, we discarded the supernatant and washed one time. After this step, the elution step for the ssDNA residue on the rGO was performed in the same manner as in the positive selection rounds.

### 4.4. Sequence Analysis and Selection of Candidates for Nonylphenol Detection

We performed a cloning process to analyze the sequence of candidates. The aptamer candidates were obtained during the final five rounds of the SELEX process. The candidates were amplified to form dsDNA, and a pET-28a vector was inserted. Each amplicon has specific sequence to BamH I and Hind III in each primer site to cut for ligation. The sequences were cut, and ligation was carried out with T4 ligase overnight at 16 °C. After the reaction, the ligation products were transformed into *Escherichia coli (E. coli)* and cultured overnight in an LB plate (lysogeny broth). DNA was extracted from the E. coli using a Plasmid Mini Extraction Kit (Bioneer, Daejeon, Korea), and sequencing was performed at Macrogen, Inc. (Seoul, Korea). The secondary structure of the candidates was confirmed by M-fold [27,28,29].

### 4.5. Gold Nanoparticle Synthesis

The gold nanoparticles used in this paper is same as it used in prior research [30,31]. Gold nanoparticle was prepared from citrate reduction of HAuCl_4_. Before use, all glassware was thoroughly washed with aqua regia (3:1 concentrated hydrochloric acid (HCl): concentrated nitric acid (HNO_3_)), rinsed 3 times with distilled H_2_O and dried in an oven at 65 °C. A 50 mL aqueous solution of HAuCl4 (1 mM, 0.02 g/50 mL) was refluxed with rapid stirring for 15 min in a 500 mL round bottom flask. And, to add quickly, sodium citrate was diluted in dH_2_O as 10 mL of 40 mM (0.1 g/10 mL). The prepared gold solution was heated to boiling point in a plate-shaped magnetic stirrer. After 20 min of boiling, the sodium citrate solution was added to the boiling HAuCl_4_ solution and stirring continued for an additional 10 min. After boiling, the color of the solution sequentially turns yellow, white, finally very dark red.

### 4.6. Characterization of the Aptamer with AuNP

To select a suitable aptamer, we performed binding affinity and specificity tests using AuNPs. To determine the binding affinity of the aptamer, it was analyzed by aggregation of the NPs. Two test lines of AuNPs were needed. For the first, 10 μL of each concentration of aptamer was reacted for 30 min with 90 μL of 5.0 nM gold nanoparticles on a 96-well polystyrene plate. For the second, 10 μL of dH_2_O was mixed with 90 μL of 5.0 nM gold nanoparticles and reacted for 30 min. Then, nonylphenol at a concentration of 100 mM was added to 0.2 μL of each counter target to obtain a final concentration of 200 nM, and the mixture was incubated for an additional 30 min. To aggregate the AuNP, 10 μL of 850 nM NaCl solution was added.

For the specificity test, the concentration of aptamer was fixed at 4 μM, and the same amounts of bisphenol A (BPA), phenol, phenylphenol (PP), diethylhexyl phthalate (DEHP), and penicillin G (PG) were used. Otherwise, the test was performed in a manner similar to that of the binding affinity test.

To confirm the detection limit of the aptamer and AuNP-based sensing system, we tested various target concentrations: 0, 0.195, 0.391, 0.781, 1.5625, 3.125, 6.25, and 12.5 nM. The aptamer concentration was 400 nM. All other conditions and processes were as stated above. The LOD value was calculated using the official formula of the International Union of Pure and Applied Chemistry (IUPAC), Equation (1):

LOD = 3 × SD/slope(1)

In this equation, SD represents the standard deviation at a minimum point, and the slope is found from the fitted graph.

### 4.7. Circular Dichroism Spectropolarimetry Analysis

Spectra obtained from circular dichroism (CD) spectropolarimetry were used to observe the conformational change of the aptamer bound to 4-n-NP [32,33]. The final aptamer concentration of 2 μM was formed using 6 μL of 100 μM aptamer (Bioneer Corp., Korea) dissolved into 264 μL of 0.1 M phosphate buffer saline buffer (PBS buffer, pH 7.4) containing 137 mM NaCl (Daejung, Korea), 2.7 mM KCl (Daejung), 10 mM Na_2_HPO_4_ (Junsei, Japan), and 2 mM KH_2_PO_4_ (Daejung). Then, 30 μL of ultrapure distilled water (DNase/RNase/Protease free, Thermo Fisher Scientific, Waltham, MA, USA) or 4-n-NP (Sigma-Aldrich, USA) were added to the solution to final concentrations of 0, 100, and 1000 nM 4-n-NP. Subsequently, the samples were incubated overnight at ambient temperature. The aptamer solution was transferred to a quartz cuvette with a 1-mm optical path length. The CD spectra were scanned from 350 to 220 nm at 25 °C using a Jasco J-1500 spectropolarimeter (Jasco Inc., Easton, MD, USA). The CD spectra were baseline (buffer only)-corrected and obtained from triplicate data accumulation.

## 5. Conclusions

Herein, we developed a nonylphenol binding aptamer through library screening and characterized this aptamer for detection of nonylphenol. Through five cycles of rGO-SELEX, an aptamer with high selectivity against other small molecules was discovered. The quantified binding affinity, shown by the value of Kd for NP7, was 194.2 ± 65.9 nM, and the LOD was 2.239 nM in the gold-nanoparticle-based system. These results show that NP7 can be used as a detection probe for nonylphenol. Additionally, its properties suggest its use for detection of NP in industrial sites or domestic fields.

The production and use of nonylphenol are regulated in many countries due to its effects on health and the environment. In the European Union, products with a nonylphenol concentration greater than 0.1% have not been allowed for distribution or use since 2003. Compared to those in similar studies, this system is adequate for detecting the target molecule (Table 1) and is more convenient. HPLC and GC/MS are the most popular and accurate detection methods for detecting EDCs in a laboratory setting. However, these methods require expensive machines and skilled technicians. The NP7 aptamer could be an integral part of an aptasensor using a signal transduction system such as gold nanoparticles, graphene nanocomposites, or quantum dots to produce a simple, cost effective, and time-saving system.

## Figures and Tables

**Table 1:** Detection methods for EDCs from previous studies.

Target	Detection Method	LOD	Reference
Nonylphenol ethoxylate	Fluorescence-based aptasensor system	696 pM	[26]
Bisphenol A	Fluorescence-based enzyme-sensor system	400 nM	[34]
Bisphenol A	AuNP-based aptasensor system	1.50 nM	[35]
Nonylphenol	Fluorescence polarization immunoassay	231 mM (= 51 mg/L)	[36]
Nonylphenol	AuNP-based aptasensor system	2.239 nM	This study

## Abbreviations

NP	Nonylphenol
4-n-NP	4-n-Nonylphenol
LOD	Limit of Detection
EDCs	Endocrine-disrupting chemicals
AuNPs	Gold nanoparticles
rGO	reduced graphene oxide
CD	Circular dichroism
SELEX	Systematic evolution of ligands using the exponential enrichment
BPA	Bisphenol A
DEHP	Diethylhexyl phthalate
PP	Phenylphenol
